# Identity status as a factor of professional self-determination in adolescence

**DOI:** 10.1192/j.eurpsy.2022.2268

**Published:** 2022-09-01

**Authors:** S. Molchanov, O. Markina, O. Karabanova, K. Kirsanov, L. Dzhepparova L

**Affiliations:** 1 Lomonosov Moscow State University, Developmental Psychology Department, Moscow, Russian Federation; 2 Financial University under the Government of the Russian Federation, Department Of Psychology And Human Resource Development, Moscow, Russian Federation; 3 Lomonosov Moscow State University, Developmental Psychology, Moscow, Russian Federation

**Keywords:** professional self-determination, personal professional perspective, identity status

## Abstract

**Introduction:**

The process of professional self-determination in adolescence is a key developmental task, the successful resolution of which determines the psychological health and well-being. Professional self-determination includes 1) the formation of a professional identity on the base of exploration the possibilities of professional choice, 2) making a decision for professional future

**Objectives:**

1) to study the features of a personal professional perspective in adolescence; 2) to reveal status of identity in the field of professional self-determination; 3) to study the relationship between the status of identity and the personal professional perspective.

**Methods:**

The modified Personal Professional Perspective technique (N.S.Pryazhnikov) and the interview to determine status identity in the professional field (D.Marsia) were used. The study involved 144 respondents aged 15 to 17 years.

**Results:**

The heterochronicity of the formation of the components of the personal professional perspective (PPP) among adolescents is revealed. The connection of the high status of identity in the professional sphere with the formation of PPP has been proved. The formation of all components of the PPP among adolescents with the status of a moratorium and status of achieved identity is higher than in group with diffusion status (U criterion, p = 0.00).

**Conclusions:**

The high status of identity in the professional sphere (moratorium and achieved identity) is associated with a high level of formation of a personal professional perspective.

**Disclosure:**

No significant relationships.

